# Prevalence and Determinants of Cardiovascular Disease Among Hypertensive Adults in Saudi Arabia: A Cross-Sectional Evaluation of Lifestyle and Clinical Risk Factors

**DOI:** 10.3390/healthcare14213668

**Published:** 2026-10-28

**Authors:** Mohammad A. Jareebi, Amal J. Alfaifi, Noor Al Khathlan, Hussam M. Shubaily, Athari Alsulami, Maysaa T. Ageel, Ghada Raja Alharbi, Maria A. Hakami, Ghazi I. Al Jowf, Farjah H. Algahtani, Faisal M. Alsalamah, Ibtihal A. Altalhi, Mohammed Abdullah Zubaylah, Ebtihal Mohammed Masrahi, Nouf Abdulelah Allehaibi

**Affiliations:** 1 Department of Family and Community Medicine, College of Medicine, Jazan University, Jazan 45142, Saudi Arabia; 2 Department of Family Medicine, Jazan Health Cluster, Jazan 45142, Saudi Arabia; ajh6666@hotmail.com (A.J.A.); maryah@moh.gov.sa (M.A.H.); 3 Respiratory Care Department, Imam Abdulrahman Bin Faisal University, Dammam 31441, Saudi Arabia; naalkhathlan@iau.edu.sa; 4 Department of Basic Medical Sciences, College of Medicine, Jazan University, Jazan 45142, Saudi Arabia; hshubaily@jazanu.edu.sa; 5 Department of Clinical Sciences, MBBS Program, Fakeeh College for Medical Sciences, Fakeeh Care Group, Jeddah 21461, Saudi Arabia; adhari.alselmi@hotmail.com (A.A.); mtageel@fcms.edu.sa (M.T.A.); gralharbi@fcms.edu.sa (G.R.A.); 6 Department of Family Medicine, Dr. Soliman Fakeeh Medical Center, Fakeeh Care Group, Jeddah 23717, Saudi Arabia; 7 Department of Public Health, College of Applied Medical Sciences, University Medical Clinics Complex, King Faisal University, Al Hofuf 37912, Saudi Arabia; galjowf@kfu.edu.sa; 8 Oncology Center, Chair of Epidemiology and Public Health Research, Faculty of Medicine, King Saud University, King Saud Medical City, Riyadh 12373, Saudi Arabia; falgahtani@ksu.edu.sa; 9 College of Medicine, Qassim University, Qassim 51452, Saudi Arabia; faisalalsalamah02@gmail.com; 10 Population Health Management Department, Jazan Health Cluster, Jazan 45142, Saudi Arabia; ebtihal2012@hotmail.com; 11 Faculty of Medicine, Batterjee Medical College, Jeddah 23819, Saudi Arabia; aleddrisi.mohammed@gmail.com; 12 College of Medicine, King Khalid University, Abha 61411, Saudi Arabia; ebtihal.masrahi@gmail.com; 13 Health Education and Health Coach, Ministry of Health, Makkah Health Cluster, Makkah 24246, Saudi Arabia; nouf.allehaibi21@gmail.com

**Keywords:** cardiovascular disease, hypertension, Saudi Arabia, diabetes mellitus, dyslipidaemia, whole grains, family history, lifestyle risk factors, inflammation

## Abstract

**Background/Objectives**: Cardiovascular disease (CVD) represents a leading source of morbidity and mortality among individuals with hypertension; however, locally relevant evidence on the determinants of CVD risk within hypertensive populations in Saudi Arabia remains limited. This study aimed to identify independent sociodemographic, lifestyle, dietary, anthropometric, clinical, and hereditary determinants of self-reported CVD among hypertensive adults. **Methods**: This cross-sectional study included 444 adults with a prior physician-confirmed diagnosis of hypertension. Cardiovascular disease (CVD) status was determined by self-report. Bivariate comparisons were performed between participants with and without CVD, followed by multivariable logistic regression to identify factors independently associated with CVD. Adjusted odds ratios (ORs) and 95% confidence intervals (CIs) were reported. **Results**: The mean age was 49 years (SD ± 15) and CVD was reported by 10.8% of participants. In bivariate analysis, CVD was significantly associated with urban residence, unemployment, larger family size, lower whole grain consumption, ex-smoking status, and the presence of diabetes mellitus and a family history of CVD. In the fully adjusted model, family history of CVD was the strongest independent predictor (adjusted OR = 17.70; 95% CI: 7.68–44.46; *p* < 0.001), followed by diabetes mellitus (adjusted OR = 5.28; 95% CI: 1.98–15.46; *p* = 0.001). Whole grain consumption was independently protective (adjusted OR = 0.34; 95% CI: 0.14–0.79; *p* = 0.016), and dyslipidaemia demonstrated a borderline positive association (adjusted OR = 2.69; 95% CI: 0.93–7.40; *p* = 0.059). **Conclusions**: Family history of CVD and diabetes mellitus were the strongest independent determinants of CVD among hypertensive adults in Saudi Arabia, while whole grain intake emerged as a practical and modifiable dietary protective factor. These findings support the integration of family-history-informed risk stratification, coordinated cardiometabolic management, and culturally adapted dietary counselling into routine hypertension care.

## 1. Introduction

Cardiovascular disease (CVD) is the leading cause of death worldwide and a major driver of global morbidity. According to the World Health Organization, an estimated 19.8 million deaths were attributable to CVDs in 2022, representing nearly one-third of all global mortality, predominantly from ischaemic heart disease and stroke [1]. This burden is substantially compounded by the ubiquity of hypertension, which remains poorly diagnosed and frequently uncontrolled. As of 2024, approximately 1.4 billion adults aged 30–79 years were estimated to have hypertension, of whom only around 23% had achieved adequate blood pressure control [2]. Hypertension should therefore be understood not only as a standalone diagnosis but as a primary pathway leading to stroke, ischaemic heart disease, heart failure, chronic kidney disease, and premature death. Identifying the determinants of CVD specifically within hypertensive populations is thus essential for delineating preventable risk clusters and informing targeted intervention. Beyond haemodynamic injury, hypertension is characterised by a state of chronic low-grade inflammation, elevated circulating pro-inflammatory cytokines, and endothelial activation, all of which contribute to accelerated atherosclerosis and heighten CVD risk in affected individuals [3].

The burden of CVD in Saudi Arabia is substantial and continues to grow. In 2021, the World Heart Federation recorded 54,791 CVD-related deaths in Saudi Arabia, with age-standardised mortality rates exceeding global averages [4]. A national household health survey estimated the prevalence of physician-diagnosed CVD among adults aged 15 years and older at 1.6%, with higher prevalence observed in males and among those aged 65 years and above [5]. These figures likely underestimate the true burden, given the inclusion of younger age groups and the reliance on self-reported diagnosis. Furthermore, despite the recognised importance of blood pressure and lipid control, significant gaps persist in the patient care pathway for hypertension and dyslipidaemia in Saudi Arabia, including suboptimal awareness, poor adherence, and inadequate disease management [6]. These findings underscore the need for studies focused specifically on hypertensive patients, who already represent a high-risk subgroup for cardiovascular events.

The determinants of CVD in hypertensive populations are multifactorial, encompassing clinical, hereditary, lifestyle, and behavioural domains. Established clinical and hereditary risk factors include advancing age, long-standing hypertension, dyslipidaemia, diabetes mellitus, obesity-related metabolic dysfunction, and family history of cardiovascular disease [2]. In a Saudi cross-sectional study among hypertensive adults, age above 45 years, diabetes, and dyslipidaemia were each independently associated with cardiovascular morbidity [7], and a separate case–control study demonstrated a significant association between dyslipidaemia and ischaemic heart disease among hypertensive Saudi patients [8]. Lifestyle factors are equally prominent: the PURE-Saudi study documented high prevalences of physical inactivity, obesity, unhealthy dietary patterns, dyslipidaemia, hypertension, and diabetes across the Saudi population [9]. However, much of the available Saudi evidence is derived from general population samples rather than specifically from patients with established hypertension. Therefore, a focused analysis of hypertensive adults is required to better characterize the factors associated with CVD in this high-risk population. This study aimed to assess the prevalence of CVD and identify clinical, sociodemographic, lifestyle, and behavioural factors independently associated with CVD among adults with physician-confirmed hypertension in Saudi Arabia.

## 2. Materials and Methods

### 2.1. Study Design and Setting

This study is a secondary analysis of a cross-sectional survey conducted in May–June 2024 across multiple regions of Saudi Arabia, the full methodology of which has been reported elsewhere [9]. In brief, the parent study examined the associations between habitual, behavioural, and dietary risk factors and hypertension among Saudi adults aged 18 years and older, using a multistage stratified random sampling design to ensure representation of diverse socioeconomic strata and urban–rural geographical regions across the Kingdom [9]. The present analysis is restricted to participants who reported a prior physician diagnosis of hypertension (*N* = 444), constituting a clinically homogeneous subgroup in whom the determinants of self-reported CVD were investigated.

### 2.2. Study Population and Eligibility Criteria

The parent study recruited Saudi citizens residing in urban and rural areas nationwide, aged 18 years and older. For inclusion in the present analysis, participants were required to report a physician-confirmed diagnosis of hypertension. Exclusion criteria for the parent study included pregnancy, severe chronic medical illness unrelated to hypertension or metabolic status, and cognitive or physical impairment precluding completion of the assessment [9]. The analytical sample for this study comprised 444 adults (13.4%) with self-reported hypertension, drawn from the 3312 parent study participants. Of the 3312 participants in the parent study, 444 (13.4%) reported a prior physician diagnosis of hypertension and constitute the analytical sample for the present study; prehypertension status was not assessed.

### 2.3. Data Collection

Data were collected via self-administered questionnaires based on validated instruments previously applied in hypertension-related research and national health surveys [10,11]. Questionnaires were developed in both Arabic and English and administered by trained medical and public health students who received standardised instruction. A pilot study involving 50 participants was conducted prior to data collection to evaluate the clarity and cultural appropriateness of the instruments [10]. Validated instruments were used for lifestyle and dietary assessments; salt intake, oral contraceptive use, and laboratory parameters (including fasting blood samples) were not collected in the parent study.

### 2.4. Measurements

#### 2.4.1. Outcome Variable

The primary outcome was self-reported physician diagnosis of CVD, coded as a binary variable (yes/no). CVD was defined broadly as any physician-confirmed cardiovascular diagnosis reported by the participant, including ischaemic heart disease, heart failure, or stroke; subtype disaggregation was not possible given the binary ascertainment.

#### 2.4.2. Sociodemographic Variables

Sociodemographic characteristics included age (continuous), sex, nationality (Saudi/non-Saudi), educational attainment (bachelor’s degree or higher vs. secondary school or lower), employment status (employed/unemployed), marital status (married/unmarried), monthly household income (<5000 SAR; 5001–10,000 SAR; >10,000 SAR), area of residence (urban/rural), housing type (owned traditional house, owned villa, owned apartment, rented), and family size (continuous).

#### 2.4.3. Anthropometric Measurements

Height and weight were self-reported. Body mass index (BMI) was calculated as weight in kilograms divided by the square of height in metres, in accordance with World Health Organization (WHO) standard procedures [12], and was retained as a continuous variable. Self-reported anthropometric values may be subject to social desirability bias, with a tendency for underreporting of weight and overreporting of height, potentially attenuating the precision of BMI estimates.

#### 2.4.4. Lifestyle and Behavioural Variables

Physical activity was assessed using validated questions adapted from the WHO STEPwise Approach to NCD Risk Factor Surveillance (STEPS) [10] and categorised as: no physical activity per week; moderate or vigorous activity for fewer than 30 min at least five times per week; and moderate or vigorous activity for at least 30 min at least five times per week, in accordance with WHO recommendations [12,13]. Smoking status was classified as never smoker, ex-smoker, or current smoker. Sleep duration was recorded in hours per night as a continuous variable.

#### 2.4.5. Dietary Variables

Dietary behaviour was assessed using five binary (yes/no) items covering: (1) consumption of whole grain products; (2) intake of five or more servings of fruits and vegetables per day; (3) selection of low-fat meat products; (4) avoidance of foods high in sugar; and (5) selection of low-fat dairy and food products.

#### 2.4.6. Health-Related Variables

Diagnoses of diabetes mellitus, dyslipidaemia, and CVD were each ascertained as binary variables (yes/no) based on self-reported physician diagnosis. Family history variables were similarly coded as binary (yes/no) for hypertension, CVD, diabetes mellitus, and dyslipidaemia.

### 2.5. Statistical Analysis

Statistical analyses were performed using R software (Version 4.2.3; R Foundation for Statistical Computing, Vienna, Austria). Continuous variables are reported as mean (standard deviation, SD) and categorical variables as frequencies and proportions. Bivariate associations between participant characteristics and CVD diagnosis were evaluated using the independent samples *t*-test for continuous variables (age, BMI, family size, and sleep duration) and the chi-squared (χ^2^) or Fisher’s exact test for categorical variables. To identify independent predictors of CVD, a multivariable binary logistic regression model was fitted, simultaneously including all variables of interest. Adjusted ORs and 95% CIs are reported. The absence of problematic multicollinearity was screened using the variance inflation factor (VIF < 5); this criterion is a necessary screen but does not confirm full model specification. Model discriminative performance was assessed using Tjur’s R2, which reflects the separation between predicted probabilities for cases and non-cases but does not assess calibration or overall goodness of fit. A two-sided *p*-value of < 0.05 was considered statistically significant.

### 2.6. Ethical Approval

Ethical approval for the parent study was obtained from the Standing Committee for Scientific Research Ethics of Jazan University (Reference No. REC-45/05/848, dated 26 November 2023). The study was conducted in accordance with the principles of the Declaration of Helsinki. All participants provided written informed consent prior to enrolment.

### 2.7. Use of Artificial Intelligence

Generative AI was used solely for language editing. Study design, data collection, analysis, and interpretation were conducted entirely by the authors.

## 3. Results

### 3.1. Participant Characteristics

A total of 444 adults with a prior physician-confirmed diagnosis of hypertension were included in the analysis. The mean age was 49 years (SD ± 15; range 18–90), and the sex distribution was approximately equal (49.1% female, 50.9% male). The vast majority were Saudi citizens (97.1%), and most resided in rural areas (66.0%). More than half held a bachelor’s degree or higher (51.6%), and 63.1% were employed. The most common housing type was an owned villa (44.4%), and the mean family size was 7.6 (SD ± 3.3). Full sociodemographic characteristics are presented in Table 1.

### 3.2. Anthropometric, Lifestyle, and Dietary Characteristics

Mean BMI was 28 kg/m^2^ (SD ± 5.3), corresponding to the overweight category. Mean sleep duration was 7.1 h/night (SD ± 1.8). Physical inactivity was prevalent, with 48.2% of participants reporting no physical activity per week, while 35.6% engaged in moderate or vigorous activity for fewer than 30 min at least five times per week. Most participants were never-smokers (73.9%). Suboptimal dietary behaviours were common: only 28.2% reported consuming five or more servings of fruits and vegetables daily, and 51.8% did not consume whole grain products. These data are summarised in Table 2.

### 3.3. Health-Related Characteristics and Comorbidity Profile

CVD was reported by 48 participants (10.8%), and 45 (10.1%) had a diagnosis of dyslipidaemia. Diabetes mellitus was highly prevalent, affecting 45.5% of the hypertensive cohort. Family history of hypertension was reported by the majority (82.9%), followed by family history of diabetes mellitus (61.9%) and family history of CVD (20.0%). Health-related characteristics are detailed in Table 3.

### 3.4. Bivariate Associations by CVD Status

Table 4 presents univariate comparisons between participants with and without CVD. Several variables were significantly associated with CVD at *p* < 0.05. Participants with CVD were more likely to reside in urban areas (52.1% vs. 31.8%; χ^2^ = 6.96, *p* = 0.008) and to be unemployed (54.2% vs. 34.8%; χ^2^ = 6.06, *p* = 0.014). Mean family size was larger among those with CVD (8.5 vs. 7.4; F = 4.53, *p* = 0.034). Whole grain consumption was significantly lower in the CVD group (33.3% vs. 50.0%; χ^2^ = 4.12, *p* = 0.042), and a higher proportion of ex-smokers was observed among CVD patients (27.1% vs. 13.9%; χ^2^ = 7.26, *p* = 0.027). Diabetes mellitus was substantially more prevalent among those with CVD (70.8% vs. 42.4%; χ^2^ = 12.81, *p* < 0.001). Family history of CVD demonstrated the strongest univariate association: 68.8% of participants with CVD reported a positive family history compared with 14.1% of those without CVD (χ^2^ = 76.29, *p* < 0.001).

### 3.5. Independent Predictors of CVD: Multivariable Logistic Regression

**Figure 1:**
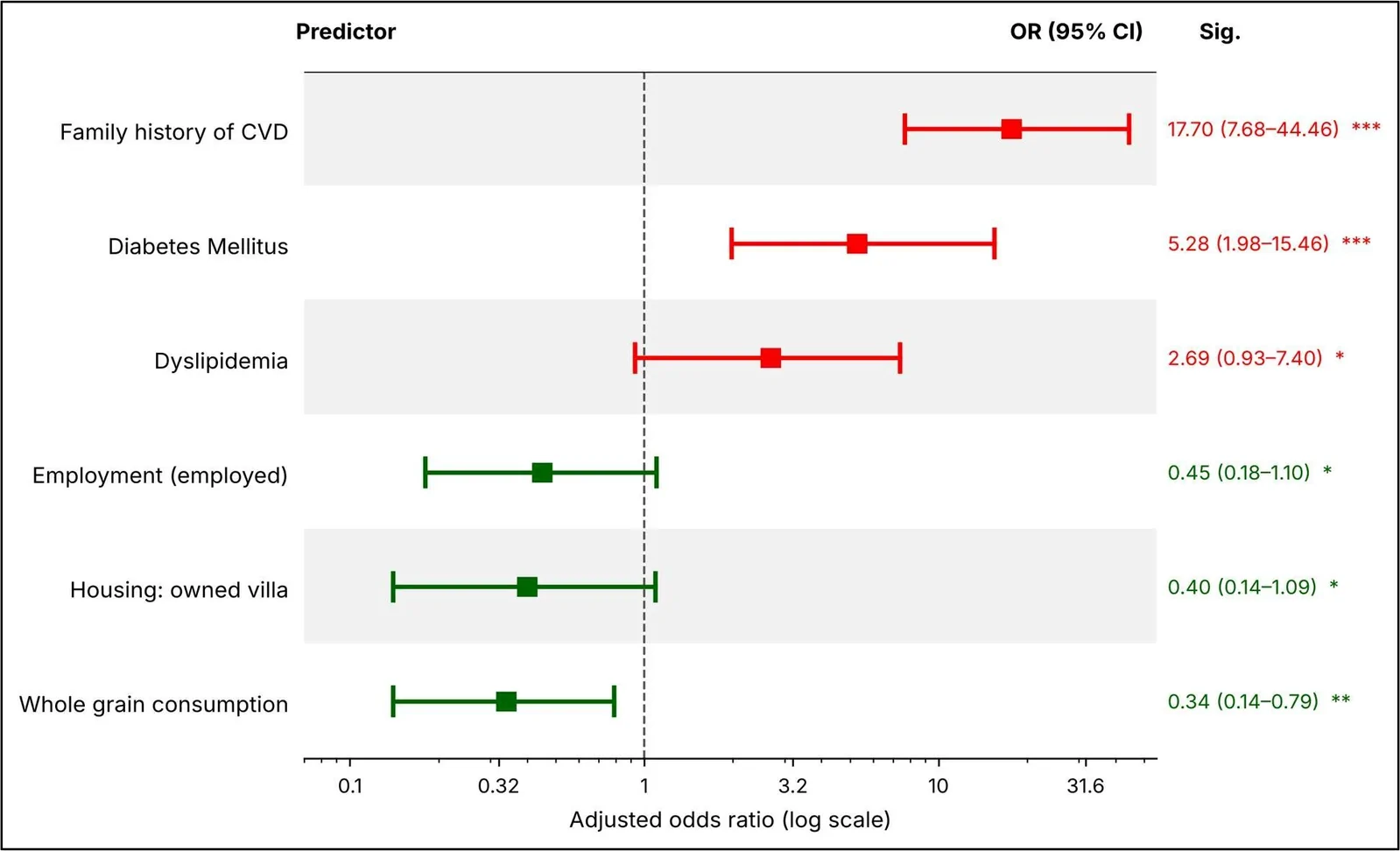
Forest plot of independent predictors of cardiovascular disease among hypertensive adults in Saudi Arabia (n = 444). Squares represent adjusted odds ratios (ORs); horizontal lines represent 95% confidence intervals (CIs). Red bars indicate increased risk (OR > 1); green bars indicate reduced risk (OR < 1). The vertical dashed line at OR = 1 represents no association. Alternating grey shading distinguishes predictor rows. Significance: * *p* < 0.05; ** *p* < 0.01; *** *p* < 0.001.

Results of the multivariable logistic regression model, adjusted for all sociodemographic, lifestyle, anthropometric, and clinical covariates, are presented in Table 5 and Figure 1. The model demonstrated good discriminative performance (Tjur’s R^2^ = 0.348). Family history of CVD was the strongest independent predictor (adjusted OR = 17.70; 95% CI: 7.68–44.46; *p* < 0.001), indicating that participants with a positive family history had approximately 18-fold higher odds of a CVD diagnosis. Diabetes mellitus was independently associated with CVD (adjusted OR = 5.28; 95% CI: 1.98–15.46; *p* = 0.001), with diabetic participants having more than five times the odds compared with non-diabetic participants. Whole grain consumption was the sole independently significant modifiable dietary factor and was associated with a 66% reduction in the odds of CVD (adjusted OR = 0.34; 95% CI: 0.14–0.79; *p* = 0.016). Dyslipidaemia demonstrated a borderline positive association with CVD (adjusted OR = 2.69; 95% CI: 0.93–7.40; *p* = 0.059). Employment status, housing type, urban residence, and family size showed borderline associations that did not reach conventional significance thresholds. Age, sex, BMI, smoking status, physical activity, and all other dietary variables were not independently associated with CVD in the fully adjusted model.

**Table 5:** Multivariable logistic regression: independent predictors of CVD among hypertensive adults (N = 444).

Predictor	Adjusted OR	95% CI	*p*-Value
**Significant independent predictors**			
Family history of CVD	17.70	7.68–44.46	<0.001
Diabetes mellitus	5.28	1.98–15.46	0.001
Whole grain consumption (yes)	0.34	0.14–0.79	0.016
**Borderline predictors (** ***p*** ** < 0.10)**			
Dyslipidaemia (diagnosed)	2.69	0.93–7.40	0.059
Employment (employed)	0.45	0.18–1.10	0.080
Housing: owned villa (ref: apartment)	0.40	0.14–1.09	0.074
Housing: rented (ref: apartment)	0.24	0.03–1.46	0.153
Urban residence	1.94	0.81–4.68	0.134
Family size	1.10	0.98–1.23	0.116
Nationality (Saudi)	0.16	0.01–2.03	0.135
**Non-significant covariates (adjusted for)**			
Age	1.00	0.97–1.03	0.999
Sex (male)	1.05	0.38–2.87	0.919
Education (secondary or below)	0.89	0.37–2.14	0.801
Marital status (married)	1.00	0.34–3.06	0.998
Income > 10,000 SAR	0.77	0.26–2.28	0.632
Income 5000–10,000 SAR	0.46	0.11–1.66	0.245
BMI	0.99	0.91–1.07	0.800
Physical activity < 30 min/session	0.65	0.25–1.65	0.365
Physical activity ≥ 30 min/session	0.99	0.30–3.08	0.990
Smoking: current smoker	0.55	0.07–2.65	0.495
Smoking: ex-smoker	1.78	0.62–5.00	0.276
≥5 fruit/vegetable servings/day	0.96	0.40–2.20	0.919
Low-fat meat choice	1.21	0.51–2.92	0.674
Avoidance of high-sugar foods	1.28	0.56–2.91	0.558
Low-fat dairy/food products	0.76	0.32–1.73	0.524
Family history of DM	0.54	0.18–1.54	0.252
Family history of HTN	1.68	0.55–5.78	0.381

Reference categories: female sex, unemployed, rural residence, never smoker, no whole grain consumption, no dyslipidaemia, no family history of CVD; housing reference: owned apartment. OR = odds ratio; CI = confidence interval. Model fit assessed using Tjur’s R^2^.

## 4. Discussion

### 4.1. Overview of Findings

This cross-sectional analysis characterised the CVD risk profile among 444 hypertensive adults in Saudi Arabia, 10.8% of whom reported a CVD diagnosis. The multivariable model demonstrated good discriminative performance (Tjur’s R^2^ = 0.348) and identified three independent predictors: family history of CVD (adjusted OR = 17.70), diabetes mellitus (adjusted OR = 5.28), and whole grain consumption (adjusted OR = 0.34, protective). Dyslipidaemia showed a borderline positive association, and several sociodemographic variables employment, urban residence, housing type, and family size exhibited effects that did not reach conventional significance. These findings reinforce that CVD risk in hypertension is embedded within a broader cardiometabolic and hereditary framework extending well beyond blood pressure alone. Hypertension itself remains substantially undertreated globally, with fewer than one in four affected adults achieving adequate blood pressure control [14]. A large, pooled analysis from the Global Cardiovascular Risk Consortium identified five modifiable factors (systolic blood pressure, non-HDL cholesterol, BMI, diabetes, and smoking) as collectively accounting for a substantial proportion of incident CVD globally [15]. Within a hypertensive cohort where all participants share elevated blood pressure as a common exposure, family history, diabetes, and carbohydrate quality emerged as the most discriminating additional determinants.

### 4.2. Non-Modifiable Risk Factors: Age, Sex, and Family History

Although participants with CVD were on average three years older (52 vs. 49 years), age was not independently significant in multivariable analysis, likely reflecting the wide age distribution, the small number of CVD events, and compression of age-related risk gradients within a uniformly hypertensive cohort. Current hypertension guidelines appropriately emphasise that clinical decisions should incorporate the totality of cardiovascular risk including comorbidities and target organ damage rather than age alone [16]. Sex was similarly non-significant, consistent with the near-equal sex distribution and the potential for under-diagnosis of CVD in women due to atypical symptom presentation.

The most striking finding was the magnitude of the family history association. Individuals with a positive family history of CVD had approximately 18-fold higher adjusted odds of a CVD diagnosis, and 68.8% of those with CVD reported such a history compared with only 14.1% of those without. Family history captures shared genetic susceptibility, common dietary environments, early-life exposures, and familial aggregation of hypertension, dyslipidaemia, diabetes, and obesity. Contemporary prevention guidelines recognise premature familial CVD as a risk-enhancing factor warranting intensified stratification [17], and recent reviews underscore its value as a cost-effective clinical screening tool [18]. While recall bias and heightened awareness of CVD in affected families may have amplified the observed association, the clinical implication is unambiguous: systematic family history documentation should function as an active trigger for earlier lipid and glucose screening, lifestyle counselling, and more frequent cardiovascular surveillance in hypertensive patients.

### 4.3. Modifiable Clinical Risk Factors: Diabetes Mellitus and Dyslipidaemia

Diabetes mellitus was the most important modifiable clinical predictor, present in 70.8% of those with CVD versus 42.4% of those without (adjusted OR = 5.28). This is consistent with the established contribution of diabetes to atherosclerotic CVD in combination with hypertension, and with the broader Saudi epidemiological context of rising cardiometabolic comorbidity [19]. The coexistence of diabetes and hypertension creates compounded vascular injury through insulin resistance, endothelial dysfunction, sympathetic activation, and atherogenic dyslipidaemia [20]. BMI was not an independent predictor; mean values of approximately 28 kg/m^2^ in both groups suggest pervasive overweight across the hypertensive cohort, attenuating between-group differentiation and supports the view that diabetes and dyslipidaemia are more proximate metabolic indicators of cardiovascular hazard than BMI alone. The 2023 ESC Guidelines for CVD in diabetes and the 2025 Standards of Care in Diabetes both underscore the importance of integrated multifactorial risk reduction encompassing blood pressure, glycated haemoglobin, lipids, and renal function, with cardioprotective glucose-lowering agents where appropriate [21,22]. The present findings add local evidence supporting coordinated hypertension–diabetes care pathways in Saudi Arabia.

A borderline positive association was observed for dyslipidaemia (adjusted OR = 2.69; *p* = 0.059). The low self-reported prevalence (10.1%) likely reflects substantial underdiagnosis or the masking effect of lipid-lowering therapy, underscoring the importance of proactive lipid screening. International lipid guidelines affirm the causal role of LDL cholesterol in atherosclerotic CVD and the primacy of lipid-lowering therapy in high-risk individuals, including those with hypertension or diabetes [23].

### 4.4. Lifestyle and Dietary Determinants

Whole grain consumption was the sole independently significant dietary predictor, associated with a 66% reduction in CVD odds (adjusted OR = 0.34). Residual confounding, reverse causation, and dietary measurement error from the binary assessment cannot be excluded, and this finding should be interpreted with caution. National health survey data for Saudi Arabia document high rates of suboptimal dietary behaviours across the adult population, including low whole grain intake [24]. Nonetheless, this association is directionally consistent with systematic reviews and meta-analyses demonstrating inverse dose–response relationships between whole grain intake and coronary heart disease, stroke, and all-cause mortality [25,26], and may reflect effects on insulin sensitivity, postprandial glycaemia, LDL cholesterol, blood pressure, and gut microbiota. In a Saudi dietary context where bread, rice, and cereal products are staple foods, whole grain substitution represents a plausible and culturally adaptable dietary target that warrants confirmation in future longitudinal studies with more detailed dietary assessment. No other dietary variable reached independent significance, though the high prevalence of suboptimal fruit, vegetable, and low-fat product consumption indicates a broader dietary risk burden that binary items may not have adequately captured.

Physical inactivity was prevalent (48.2%) but not independently associated with CVD, potentially reflecting self-report misclassification or reverse causality following diagnosis. Similarly, ex-smoking was more common in the CVD group (27.1% vs. 13.9%) in bivariate analysis consistent with behavioural change post-diagnosis but did not reach significance in the adjusted model. Both physical activity and tobacco cessation retain well-established mechanistic importance in CVD prevention and should remain core components of hypertension management irrespective of their statistical performance in this cross-sectional sample [27,28].

### 4.5. Sociodemographic Context and Public Health Implications

Urban residence, unemployment, and larger family size were each associated with CVD in bivariate analysis, but none reached significance in the adjusted model. Employment showed a non-significant inverse association in the adjusted model (adjusted OR = 0.45; *p* = 0.080); because the confidence interval includes the null, no protective effect can be inferred from this finding. The urban CVD excess in bivariate analysis is consistent with the dietary transition, physical inactivity, and obesogenic environments accompanying rapid urbanisation in Saudi Arabia. The social determinants of CVD encompassing economic stability, neighbourhood context, and healthcare access are increasingly recognised as fundamental risk drivers [29], and these findings emphasise that pharmacological and clinical management must be complemented by social and environmental prevention strategies.

Three clinical implications follow from this analysis. Family history of CVD should be systematically documented at hypertension diagnosis and used to trigger earlier, more intensive cardiometabolic screening. Diabetes in hypertensive patients should be managed through integrated, protocol-driven pathways addressing blood pressure, glycaemia, lipids, and renal function simultaneously. Lipid screening should be proactive and universal rather than symptom-driven, given the likely underdiagnosis of dyslipidaemia in this population. At the public health level, CVD prevention programmes in Saudi Arabia should incorporate culturally appropriate nutrition education emphasising whole grain and dietary fibre intake alongside established messages on sodium reduction, weight management, physical activity, and smoking cessation in alignment with Saudi Vision 2030 health objectives [30].

### 4.6. Strengths and Limitations

This study’s principal strengths include its exclusive focus on a clinically defined hypertensive cohort, the multidimensional assessment of risk domains, and the use of multivariable regression to adjust for a broad range of potential confounders. Key limitations include the cross-sectional design, which limits causal inference and is susceptible to reverse causation; reliance on self-reported variables with attendant risks of recall and social desirability bias; the absence of objective clinical parameters (measured blood pressure, lipid profiles, HbA1c, waist circumference, and medication data); and the relatively small number of CVD events (n = 48), which limited statistical power for several predictors. CVD was not subtyped by clinical entity, and dietary assessment was restricted to five binary items that do not capture dietary pattern, sodium intake, or food processing. Generalisability is limited to hypertensive adults who self-reported a diagnosis and may not extend to those with undiagnosed or poorly accessible hypertension. Future studies should employ longitudinal designs, incorporate objective clinical measurements, enable CVD subtype analyses, and evaluate the impact of targeted dietary and risk stratification interventions in this population. The ratio of CVD events to model covariates raises concern for potential overfitting and unstable coefficient estimates; the regression findings should therefore be regarded as exploratory and hypothesis-generating rather than confirmatory. Improving hypertension detection and control in Saudi Arabia remains a priority aligned with global targets [31].

## 5. Conclusions

Among hypertensive adults in Saudi Arabia, CVD was independently associated with hereditary, clinical, and dietary determinants. Family history of CVD was the strongest independent predictor, highlighting the clinical importance of systematic familial risk assessment in routine hypertension management. Diabetes mellitus was also strongly and independently associated with CVD, reinforcing the need for integrated cardiometabolic care in this population. Whole grain consumption emerged as the sole independently significant protective dietary factor, suggesting that carbohydrate quality warrants greater emphasis in dietary counselling for hypertensive patients. Dyslipidaemia demonstrated a clinically plausible borderline association, supporting proactive lipid screening. Although age, sex, BMI, smoking, and physical inactivity were not independently significant in this analysis, they retain established clinical relevance in comprehensive cardiovascular prevention. These findings support a family-history-informed risk stratification approach, integrated diabetes and lipid management, and culturally adapted lifestyle interventions in the hypertensive Saudi population. Given the cross-sectional design, causal inferences cannot be drawn, and longitudinal studies are required to validate these associations.

## Figures and Tables

**Table 1:** Sociodemographic characteristics of study participants (N = 444).

Variable	*n*	% or Mean (SD)
**Age** (years)	444	49 (15)
**Sex**	444	
Female	218	49.1%
Male	226	50.9%
**Nationality**	444	
Saudi	431	97.1%
Non-Saudi	13	2.9%
**Education**	444	
Bachelor’s degree or above	229	51.6%
Secondary school or below	215	48.4%
**Employment status**	444	
Employed	280	63.1%
Unemployed	164	36.9%
**Marital status**	444	
Married	337	75.9%
Unmarried	107	24.1%
**Monthly income**	444	
<5000 SAR	116	26.1%
5000–10,000 SAR	87	19.6%
>10,000 SAR	241	54.3%
**Area of residence**	444	
Rural	293	66.0%
Urban	151	34.0%
**Housing type**	444	
Owned villa	197	44.4%
Owned traditional house	118	26.6%
Owned apartment	88	19.8%
Rented	41	9.2%
**Family size**	444	7.6 (3.3)

Abbreviations: *n*: sample size; SD: standard deviation; BMI: Body Mass Index; SAR: Saudi Arabian Riyal. USD equivalents are approximate, based on an exchange rate of 1 SAR = 0.267 USD.

**Table 2:** Anthropometric, lifestyle, and dietary characteristics of study participants (N = 444).

Variable	*n*	% or Mean (SD)
**Height** (cm)	444	163 (9.3)
**Weight** (kg)	444	73 (15)
**BMI** (kg/m^2^)	444	28 (5.3)
**Sleep duration** (hours/night)	444	7.1 (1.8)
**Physical activity level**	444	
No physical activity per week	214	48.2%
Moderate/vigorous activity < 30 min, ≥5 times/week	158	35.6%
Moderate/vigorous activity ≥ 30 min, ≥5 times/week	72	16.2%
**Smoking status**	444	
Never smoker	328	73.9%
Ex-smoker	68	15.3%
Current smoker	48	10.8%
**Dietary behaviour**		
Whole grain product consumption (Yes)	214	48.2%
≥5 servings of fruits and vegetables/day (Yes)	125	28.2%
Low-fat meat products (Yes)	277	62.4%
Avoidance of high-sugar foods (Yes)	204	45.9%
Low-fat dairy and food products (Yes)	173	39.0%

**Table 3:** Health-related characteristics of study participants (N = 444).

Variable	*n*	% or Mean (SD)
**CVD**	444	
Yes	48	10.8%
No	396	89.2%
**Diabetes mellitus (DM)**	444	
Yes	202	45.5%
No	242	54.5%
**Dyslipidaemia**	444	
Yes	45	10.1%
No	399	89.9%
**Family history of DM**	444	
Yes	275	61.9%
No	169	38.1%
**Family history of hypertension (HTN)**	444	
Yes	368	82.9%
No	76	17.1%
**Family history of CVD**	444	
Yes	89	20.0%
No	355	80.0%
**Family history of dyslipidaemia**	444	
Yes	43	9.7%
No	401	90.3%

**Table 4:** Bivariate analysis of factors associated with CVD diagnosis among hypertensive patients (N = 444).

Variable	No CVD (*n* = 396) *n*	%	CVD (*n* = 48) *n*	%	*p*-Value
**Sociodemographic**					
Age, mean (SD)	49 (14)		52 (19)		0.183
Sex: male	198	50.0	28	58.3	0.348
Employment: employed	258	65.2	22	45.8	**0.014**
Residence: urban	126	31.8	25	52.1	**0.008**
Married	301	76.0	36	75.0	0.990
Income > 10,000 SAR	212	53.5	29	60.4	0.580
**Anthropometric and habitual**					
BMI, mean (SD)	28 (5.2)		28 (5.6)		0.818
Family size, mean (SD)	7.4 (3.1)		8.5 (4.2)		**0.034**
Sleep hours, mean (SD)	7.1 (1.8)		7.1 (1.6)		0.797
**Lifestyle and dietary**					
Whole grain consumption: yes	198	50.0	16	33.3	**0.042**
≥5 fruit/vegetable servings: yes	110	27.8	15	31.3	0.738
Low-fat meat: yes	245	61.9	32	66.7	0.624
Avoidance of high-sugar foods: yes	179	45.2	25	52.1	0.453
Low-fat products: yes	154	38.9	19	39.6	0.964
**Smoking status**					0.027
Current smoker	46	11.6	2	4.2	
Ex-smoker	55	13.9	13	27.1	
Never smoker	295	74.5	33	68.8	
**Physical activity**					0.505
No physical activity	190	48.0	24	50.0	
<30 min, ≥5 times/week	144	36.4	14	29.2	
≥30 min, ≥5 times/week	62	15.7	10	20.8	
**Health-related**					
Diabetes mellitus: yes	168	42.4	34	70.8	**<0.001**
Dyslipidaemia: yes	36	9.1	9	18.8	0.066
Family history of CVD: yes	56	14.1	33	68.8	**<0.001**
Family history of DM: yes	240	60.6	35	72.9	0.133
Family history of HTN: yes	328	82.8	40	83.3	0.990
Family history of dyslipidaemia: yes	35	8.8	8	16.7	0.140

Values are *n* (%) unless otherwise stated. Continuous variables were compared using the two-sample *t*-test; categorical variables were compared using the chi-squared (χ^2^) or Fisher’s exact test (Bold denotes statistically significant results (*p* < 0.05). CVD = cardiovascular disease; DM = diabetes mellitus; HTN = hypertension; BMI = body mass index.

## Data Availability

The datasets generated and analyzed during the current study are available from the corresponding author upon reasonable request. Data are not publicly available due to informed consent restrictions and the risk of participant re-identification.

## Abbreviations

The following abbreviations are used in this manuscript:

**Abbreviation**	**Definition**
AI	Artificial Intelligence
BMI	Body Mass Index
CI	Confidence Interval
CVD	Cardiovascular Disease
DM	Diabetes Mellitus
HTN	Hypertension
OR	Odds Ratio
SAR	Saudi Arabian Riyal
VIF	Variance Inflation Factor
WHO	World Health Organization
